# Clinical problem selection for machine learning-based clinical decision support in the intensive care unit: complexity, actionability, and the way forward

**DOI:** 10.3389/fmed.2026.1734400

**Published:** 2026-02-17

**Authors:** Anirudh Vinnakota, Matthew Hodgman, Daniel Ehrmann

**Affiliations:** 1 Department of Pediatrics, Division of Cardiology, University of Michigan Medical School, Ann Arbor, MI, United States; 2 Department of Computational Medicine and Bioinformatics, University of Michigan Medical School, Ann Arbor, MI, United States

**Keywords:** artificial intelligence, clinical decision support, Information Value Chain Theory, intensive care unit, machine learning

## Abstract

Machine learning (ML)-based clinical decision support (CDS) in the intensive care unit (ICU) has the potential to improve medical decision-making and patient outcomes. The chasm between model development and bedside deployment threatens these outcomes. Drivers of the chasm are multifactorial and have been extensively studied. This perspective focuses on a critical phase of the ML pipeline that contributes to the chasm: problem selection. Problem selection is a challenging exercise requiring engagement of the entire multidisciplinary ML team, but there lacks a practical framework to guide discussions in a way that leads to meaningful candidate problem evaluation. We propose specific questions informed by the Information Value Chain Theory and other empirical groundings to consider while performing problem selection. The questions are focused on complexity and actionability and operationalized into a complexity-actionability problem evaluation (CAPE) checklist usable by ML teams to determine if the candidate problem-CDS pair is poised for impact or requires reformulation. We conclude by looking to the future where more effective CDS is routinely deployed to the bedside, while also suggesting that optimizing the execution of care in parallel with CDS is critical to achieve maximum value of the technology to bedside information and meaningful, scalable improvements in patient outcomes.

## 1 Introduction

The “chasm” between the development of machine learning (ML)-based clinical decision support (CDS) systems and their deployment at the intensive care unit (ICU) bedside is wide (1). The problem is multifactorial, with known gaps in data quality (2), validation (3), usability (4), governance (5), and sociotechnical integration (6, 7). However, while addressing those gaps are important, this article focuses on an underspecified determinant of deployment success: choosing the right clinical problem in the first place (8).

The current paradigm for optimal ICU problem selection is poorly defined, which may contribute to failed clinical deployments. This perspective presents an empirically informed practical framework for ML teams on *how* to evaluate clinical problems for ML-based CDS. The framework relies upon known stages of Information Value Chain Theory (IVCT) which conceptualizes how technology use translates to patient outcomes through successive stages of information processing, decision-making, and care actions (9). Since performance through the stages of IVCT impacts the effectiveness of CDS (10), we argue for the importance of optimal problem selection as an upstream driver of value for the entire chain. We provide a checklist to help ML teams identify optimal problems for CDS, with associated empirical groundings in IVCT and related literature, operationalized definitions, and illustrative examples.

## 2 From data to information to decision: choosing a problem with optimal complexity

The early stages of the IVCT in a CDS paradigm require interaction with CDS to translate data to information needed to change a decision. Whether that translation is value additive depends critically on the complexity of the target clinical problem. Problem complexity has been defined multidimensionally along domains of system element interrelatedness (i.e., competing medical problems and priorities), dynamism (i.e., magnitude and rate of change of medical problems), emergence (i.e., the additive effect of individual medical problems is greater than their isolated effects), non-linear system behavior (i.e., unpredictability in rate of change of problems or response to treatment), and other domains (11). In the ICU, physicians rely on “bounded rationality,” using heuristics and approximations of patient states to manage high complexity decisions (12, 13). In high complexity circumstances, approximations are associated with significant uncertainty (14) and in low complexity circumstances, approximations are associated with very low uncertainty. The ability of CDS to provide additive value through an IVCT-based paradigm can be compromised, as described using examples in the subsequent sections.

### 2.1 When CDS cannot add sufficient value to the information value chain because the problem is too complex

We illustrate this complexity-based evaluative process using a longitudinal theme of decision-making related to mechanical ventilation in the ICU. We choose the example of mechanical ventilation because it is a prototypical use case in the ICU that generates problems (and decisions) with a broad range of complexity depending on the clinical context. This example allows us to keep the general use case fixed while varying problem complexity to illustrate the relationship between complexity to the value add of CDS within IVCT.

Imagine a patient who is mechanically ventilated after cardiac surgery. An ML team is building CDS that helps determine an optimal ventilator parameter titration strategy. The CDS will use a reinforcement learning paradigm to recommend specific changes to ventilator parameters designed to reduce 90-day mortality. In this example, the *complexity* of the decision-making (i.e., ventilator titration in acute dynamic illness) is high. Furthermore, the *complexity* of the relationship between actions (ventilator weans) and outcomes the system is designed to optimize (90-day mortality) is very high. In fact, the degree of complexity imparts risk of CDS failure on its own accord. Specifically, in a high complexity problem, CDS cannot generate enough value to translate data to information in a way that meaningfully augments the decision-making. There are several mechanistic underpinnings. For example, ventilator titration in practice must incorporate *complex* factors that are not or cannot be encoded in training data. Competing priorities (e.g., minimizing metabolic demand), co-morbidities (e.g., congestive heart failure) system interrelatedness (e.g., cardiopulmonary interactions), dynamic changes in respiratory system compliance, and the treatment team’s risk tolerance are some of many factors that create a decision-making milieu for which CDS may not—or cannot—be helpful. Furthermore, measuring the system’s success in clinical practice is challenging. What is the ground truth against which the system will be evaluated to determine if a predicted action (i.e., ventilator wean) was “good” given the clinical context (reduction of 90-day mortality is not acutely helpful)? Imparting CDS into a complex clinical problem—one where bounded rationality leads to uncertain approximations of the true patient state—might foster CDS *distrust* from the clinical team as being overly reductive, as has been reported in other use cases previously (15). When faced with a clinical problem that is overly complex, ML teams should consider focusing on a different, less complex problem that is well-encoded in the training data and whose success can be easily measured in a time horizon that is clinically meaningful. This may involve decomposing the original clinical problem to evaluable subtasks (e.g., re-intubation risk after standardized SBT) within short outcome horizons (≤24–48 h) and high label reliability.

### 2.2 When CDS cannot add sufficient value to the information value chain because the problem is not complex enough

Imagine the ML team shifts toward CDS that flags malpositioned endotracheal tubes on standard chest X-rays. The system will use a convolutional neural network-based approach trained using a corpus of prior chest X-rays labeled by expert clinicians (the label is tube distance from carina in millimeters [mm], and a malpositioned endotracheal tube was defined as tube distance <10 mm from the carina). In this example, the *complexity* of the decision-making (i.e., determination of endotracheal tube malposition) is low. In fact, the degree of complexity (and specifically, lack thereof) imparts risk of CDS failure on its own accord. In a low complexity problem, CDS cannot generate enough value to translate data to information in a way that meaningfully augments decision-making because there is fundamentally no need for augmentation in the setting of low uncertainty. Determining a malpositioned endotracheal tube on chest X-ray is done routinely without significant difficulty or latency by bedside providers. When clinicians already act within minutes with high accuracy, additional alerts create false-work (verification without clinical gain). Even worse, when the model inevitably generates a prediction that is quickly verified as wrong, resentment or frustration may ensure. Overall, imparting CDS into a low complexity clinical problem, one where the decision-making is easy and routinely performed well, might make the clinical team *discount* CDS as non-additive to their decision-making. Wrong predictions in this circumstance, which will occur in any model, are kryptonite to CDS success because clinical users already skeptical about the value add will have unambiguous data to support the narrative. When faced with a clinical problem that risks low complexity, ML teams should consider focusing on a more complex, “pain point” related to the original problem or scope-tighten to scenarios where the task is genuinely hard. For example, instead of CDS predicting endotracheal tube malpositioning on daily chest X-ray in adults, the team might pivot to predicting endotracheal tube malpositioning immediately following intubation in neonates where the prediction of tube malpositioning is more difficult given smaller patient size and room for error.

### 2.3 The “goldilocks” principal

As illustrated in the hypothetical examples above, choosing a problem ripe for CDS requires an early evaluation of its clinical complexity. There is limited empirical evidence examining the real-world utility of ML-based CDS systems, with much of the literature demonstrating the utility of rule-based CDS. Previous studies demonstrate that rule-based CDS utilized for more complex decision making are used less frequently by the clinical team—often since there is more clinician distrust in such CDS tools (9, 12, 16). Conversely, problems with low complexity risk CDS failure because providers are likely to *discount* model predictions with low perceived added value. Therefore, ML teams should focus on identifying use cases with optimal complexity where CDS has the ability to translate data into information in a way that meaningfully adds value to the decision-making process. In other words, teams should aim for the “goldilocks” zone of medical complexity.

### 2.4 How ML teams can practically identify a “goldilocks” problem for maximally valuable CDS

We propose three key questions for ML teams to ask related to complexity as part of a broader problem evaluation framework. First, can we define a single decision and its success metric in ≤1 sentence? Second, is the success metric itself identifiable and measurable within a clinically meaningful horizon, with sufficient reliability to judge whether the decision was “good”? Third, is the problem complex enough to create clinician uncertainty yet structured enough that a CDS recommendation would reduce uncertainty rather than add noise? Here, we define uncertainty using Bhise’s definition as “a subjective perception of an inability to provide an adequate explanation of the patient’s health problem (14).” Uncertainty in this context can include either aleatoric uncertainty (uncertainty from random variability) or epistemic uncertainty (uncertainty from incomplete knowledge), or both (17). We define noise as “unwanted random variability in decision making without improvement in decision-making quality,” in this context secondary to flawed CDS (18, 19).

We encourage ML teams to reach answers to these questions by consensus, and when consensus cannot be reached, to gather empirical evidence to support responses. For example, providing case vignettes to potential CDS end-users and asking them to rate their level of uncertainty with regards to a specific problem and associated decision may be illuminating. Providing sample CDS outputs and gauging the impacts on that uncertainty may help reveal a tendency toward value-add or noise. Operationally, all three items must receive a “yes” from the ML team in order for a candidate clinical problem to “pass.” Items receiving “no” or “maybe” should prompt additional discussion to determine how or if they can be resolved.

## 3 Choosing a problem with CDS actionability

The later stages of the IVCT in a CDS paradigm require a decision that results in an action capable of meaningfully altering care. As such, choosing a problem with “goldilocks” complexity is necessary but insufficient to maximize value in an IVCT paradigm. As we described previously, problems must also map to a CDS prediction that is *actionable* (20). Actionability transcends the additive *awareness* made possible by a CDS prediction. Actionability measures the degree to which awareness translates to a definable action, executable in a short time horizon relative to the model’s prediction, that was not previously considered or prioritized using clinical judgment alone. Imagine a patient who is in the ICU 1 week following diagnosis of septic shock. The patient has recovered from the initial shock and is recovering as expected. An ML team is interested in building CDS that predicts need for intubation in critically ill patients. The team applied a recurrent neural network to time series data to predict the need for intubation in the next 48 h. The system is operationalized to send an alert to the care team when the predicted probability of intubation is greater than 50 percent. In this example, problem selection along the complexity domain is reasonable. The problem and its success metric are easily definable in one sentence (preventing late recognition of respiratory decompensation to avoid intubation), the success metric itself identifiable and measurable within a clinically meaningful horizon (avoiding intubation in the next 48 h), and the problem has “goldilocks” complexity (the decision-making is complex enough to generate genuine clinical uncertainty that may be effectively reduced with CDS).

However, the CDS may struggle with an actionability problem. Consider the hypothetical scenario where the treatment team’s approximation of respiratory failure risk is low. The team receives an alert that the patient is predicted to require intubation in the next 48 h. A provider examines the patient, who looks well, reinforcing the pre-existing approximation. Even if the model has *near perfect* measures of performance, what is the care team supposed to do? The patient is already monitored closely in the ICU with vital signs acquired every hour, escalation of respiratory support is not indicated on clinical grounds even if predicted to occur with near certainty, and obtaining additional lab work or imaging studies without a clinical indication utilizes unnecessary resources while being unlikely to change the team’s pre-existing approximation of the patient’s clinical state (which is already informed by serial laboratory, imaging, and clinical assessment data). The CDS is therefore not actionable in *this* circumstance.

### 3.1 How ML teams can practically identify an actionable problem for maximally valuable CDS

We propose three key questions related to actionability as part of a broader problem formulation framework, which now forms the complete complexity-actionability problem evaluation (CAPE) checklist (Table 1). First, is there a definable action that can be taken within the clinically appropriate time frame after the model’s prediction that is plausibly linked to an improved outcome? Second, do CDS alerts prompt actions that would not otherwise occur based on the care team’s interpretation of readily available data alone? Third, is the expected rate of non-actionable alerts below the care-team and unit tolerance? These criteria map to existing empirical constructs. The first item is derived from IVCT, which requires that CDS predictions meaningfully change downstream decisions and care processes rather than simply improve diagnostic discrimination in isolation. The second item is based on our prior actionability work (20), in which actionable CDS must elucidate care pathways (additional diagnostics, different treatment plan, or meaningful change in clinical monitoring/surveillance) that were not previously known given the existing clinical data without CDS. In other words, it must result in an entropic reduction of the subsequent diagnostic and/or therapeutic probability distributions such that decision-making becomes clearer. The third item aligns with the alert-fatigue literature (not exclusively limited to CDS), where persistently high override or ignore rates (often in the 70–80% range) are interpreted as evidence of poor actionability and reduced safety pathways (21, 22). Though the elements of the checklist have empirical basis, multidisciplinary discussion is required to decide whether to pursue the candidate problem through the lens of actionability. We encourage teams to (a) envision a range of clinical scenarios, and their respective prevalences, in which the proposed CDS might be used (b) estimate the frequency of violations of the three item checklist to determine an estimated ratio of actionable/inactionable alerts, and then (c) discuss whether that ratio is acceptable given the clinical and care context, for example along the domains of resource utilization, workflow disruption, and/or model trust. When ambiguity exists and consensus among the ML team is unable to be reached, empirical study (for example, using different scenarios in the simulation laboratory) might help to answer these critical questions. All three items in the actionability section of the CAPE checklist must receive a “yes” from the ML team for a candidate clinical problem to “pass,” with non-yes answers requiring further discussion and/or problem reformulation. To illustrate how the CAPE checklist can be used with the three mechanical ventilation examples previously introduced, see Supplementary Tables 1–3.

**Table 1:** The complexity-actionability problem evaluation (CAPE) checklist.

Domain	Question
Complexity	Can we define one clinical decision and its success metric in ≤1 sentence?
	Is the success metric itself identifiable and measurable within a clinically meaningful horizon (e.g., within the same shift/decision window), with sufficient reliability to judge whether the decision was “good”?
	Is the problem complex enough to create clinician uncertainty yet structured enough that a CDS recommendation would reduce uncertainty rather than add noise?
Actionability	Is there a definable action that can be taken within the clinically appropriate time frame after the model’s prediction that is plausibly linked to an improved outcome?
	Do CDS alerts prompt actions that would not otherwise occur based on the care team’s interpretation of readily available data alone?
	Is the expected rate of non-actionable alerts below the care team and unit’s tolerance?

## 4 Discussion

**Figure 1:**
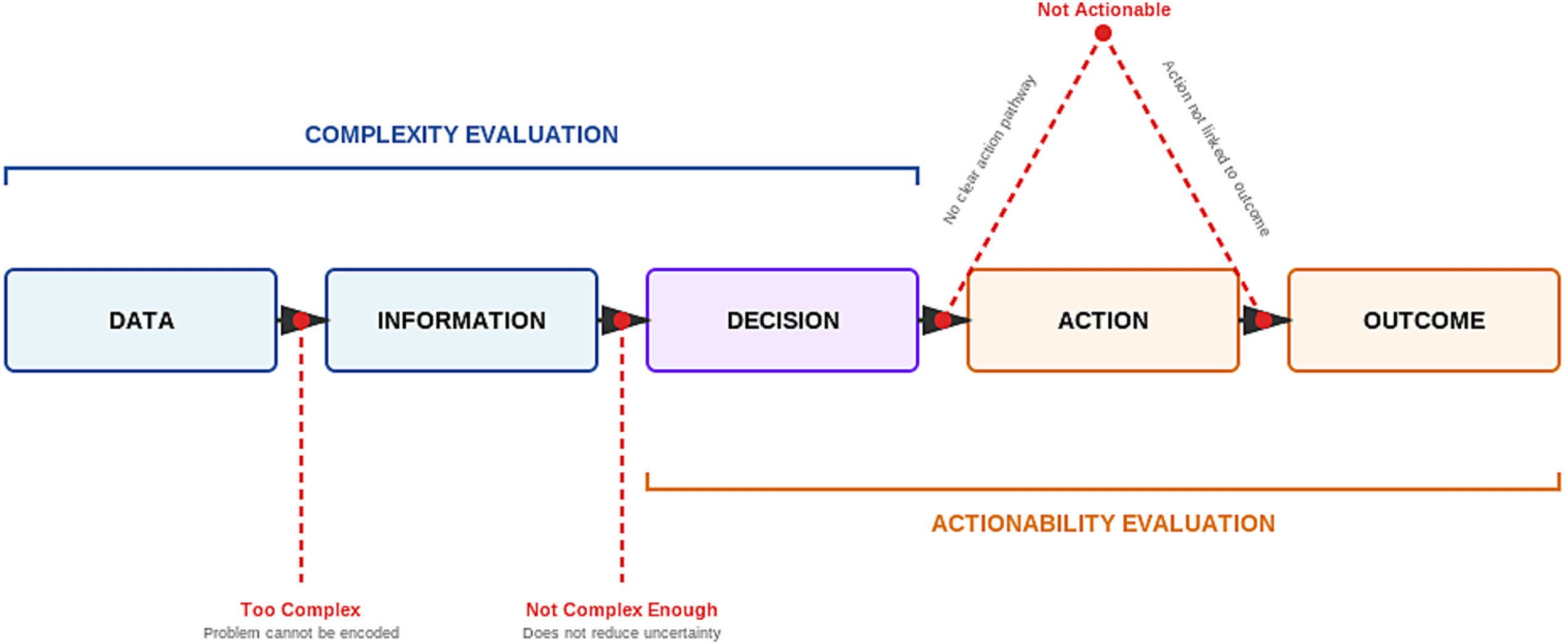
The Information Value Chain Theory (IVCT) framework showing where complexity and actionability evaluations impact clinical decision support (CDS) success. Complexity determines whether CDS can effectively translate data to information and reduce decision uncertainty (early IVCT stages), while actionability determines whether improved decisions translate to meaningful care actions and outcomes (later IVCT stages). Problems that are too complex fail at data-to-information translation, problems that lack sufficient complexity fail to reduce decision uncertainty, and problems that lack actionability fail to translate decisions into meaningful actions that impact outcomes. Candidate problems for machine learning-based CDS that are in the ‘Goldilocks zone’ of complexity and optimally actionable can successfully traverse the entire value chain to improve patient outcomes.

Clinical problem selection along the domains of complexity and actionability for CDS destined for use in the ICU is critical. While much has been written about CDS development (23), the current guidance for CDS problem selection lacks practicality and simplicity. We attempted to fill that gap by providing a checklist for teams to consider when evaluating a clinical problem’s complexity and actionability, embedded within the established IVCT framework (Figure 1). We encourage teams to publish their CAPE checklist evaluation methods and results as supplemental material accompanying CDS reports.

However, we worry that simply bringing CDS focused on better problems to the bedside is necessary but insufficient to change patient outcomes. Indeed, few CDS systems have improved patient outcomes in randomized controlled trials (24). A root cause has been focusing solely on clinical decision *support* without complimentary clinical *execution* support (CES). We define CES systems as those that semi-automate the titration of treatments through closed-loop control systems under the supervision of the clinician team. CDS focuses on the “what” of care (e.g., “what’s the diagnoses? What’s the best treatment?) elucidated through ML or rules-based algorithms that ingest clinical data and predict a class or risk among candidate outputs (10, 25, 26). In contrast, CES focuses on the “how” of care elucidated through ML or mathematical algorithms that predict a treatment change to achieve treatment goals in a closed loop paradigm (27). If CDS were perfect and promoted better treatment decisions earlier, ICU clinicians may still be left to execute those treatments in variable ways heavily impacted by personal and institutional biases and field-specific dogma (28).

**Figure 2:**
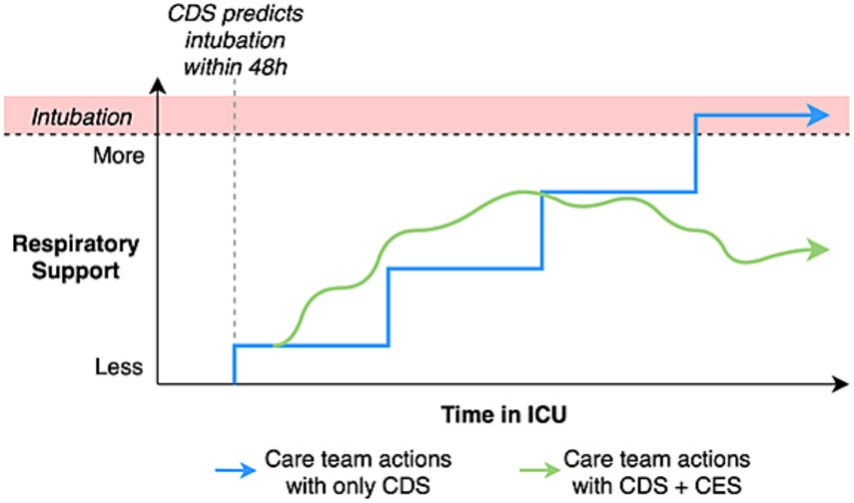
A clinical decision support (CDS) + clinical execution support (CES) complementary framework may improve patient outcomes by delegating different problems to clinicians using CDS vs. CES loop systems with human oversight. The CDS graph is depicted as stepwise increments signifying discrete, idiosyncratic decision points, whereas CES is shown as more frequent goal-directed changes representing the real-time adjustments offered by feedback within a closed loop paradigm.

Using the intubation predictor example above, if the CDS worked perfectly and identified a patient in early respiratory failure, not previously known to the care team, resulting in an early escalation of respiratory support, this would likely be deemed a success of the system through a *decision* support lens. However, the *execution* of respiratory support escalation (i.e., what to escalate the patient to, when to re-evaluate, what threshold to use to further escalate) is likely to be idiosyncratic, contributing to poor outcome regardless. The utilization of CES, as a complement to CDS, may help optimize value of the IVCT specifically between action and outcome. For example, a clinician might escalate to non-invasive positive pressure ventilation (NIPPV), set initial settings and a target range for a work of breathing surrogate [e.g., rapid shallow breathing index (29)], and enable a closed loop CES to modulate NIPPV to maintain the patient in the desired range. An example of how this system might work is shown in Figure 2.

Thus, we envision a future of both CDS and CES working together—CDS that defines the *what* of care (what is the patient’s diagnosis, what is the best next treatment) and CES to define the *how* of care (how to titrate selected therapies toward specified goals), with the human-in-the loop. Problem selection through the lens of complexity, actionability, and IVCT is relevant for both, and united in a goal to maximally improve the outcomes of critically ill patients that need it most.

## Data Availability

The original contributions presented in the study are included in the article/Supplementary material, further inquiries can be directed to the corresponding author.
