# Supplementary material for: Clinical problem selection for machine learning-based clinical decision support in the intensive care unit: complexity, actionability, and the way forward
Source: Front Med (Lausanne). 2026 Feb 17;13:1734400. doi: 10.3389/fmed.2026.1734400 (PMC12953083; doi:10.3389/fmed.2026.1734400)
Supplement: Supplementary file 1 [file Table_1.docx]

| **Supplemental Table 1: Example of an Overly Complex Problem, Using the CAPE checklist** | | |
| --- | --- | --- |
| **Clinical Problem: Using reinforcement learning to recommend ventilator parameter changes to reduce 90-day mortality in post-cardiac surgery patients** | | |
| **Checklist Item - Complexity** | **Assessment** | **Pass/Fail** |
| Clinical decision and success metric ≤1 sentence | Optimize ventilator parameter settings to reduce a patient’s 90-day mortality. | Pass |
| Success metric measurable within meaningful horizon | No. 90-day mortality cannot be evaluated in real-time by the clinical team and there is no opportunity for feedback for immediate decision validation. The ground truth for whether a ventilator wean was appropriate is unclear. | Fail |
| Recommendation reduces uncertainty without adding noise | This problem is too complex. There are too many unmeasurable factors. CDS-determined decisions will add to uncertainty, both aleatoric and epistemic, due to complex interplay between ventilation titration and 90-day mortality, system interrelatedness, and patient comorbidities affecting mortality. This would add noise instead of reducing uncertainty. | Fail |
| **Checklist Item - Actionability** | **Assessment** | **Pass/Fail** |
| Action with an appropriate time frame? | Yes, the algorithm predicts specific parameter changes that can be quickly executed by the care team. | Pass |
| Alerts prompt action that otherwise would not occur | This is uncertain. Experienced clinicians may already titrate ventilator settings using their clinical expertise and judgment which would incorporate several factors that the CDS cannot adequately capture. | Fail |
| Non-actional alert rate is below the care team’s tolerance | High, given the likely mismatch between CDS recommendations and clinical reality. There is a significant risk of clinician distrust and override. | Fail |
| **OVERALL** | **Fail - Problem is too complex with inadequate variable capture and success metrics that are too distant to be clinically meaningful** | |

| **Supplemental Table 2: Example of a Problem Not Complex Enough, Using the CAPE Checklist** | | |
| --- | --- | --- |
| **Clinical Problem: Using machine learning to flag malpositioned endotracheal tubes on routine portable adult chest X-rays** | | |
| **Checklist Item - Complexity** | **Assessment** | **Pass/Fail** |
| Clinical decision and success metric ≤1 sentence | Detect malpositioned endotracheal tubes on chest X-ray that are < 10 mm from the carina to prompt repositioning | Pass |
| Success metric measurable within meaningful horizon | Yes, tube position can be verified immediately by the clinical team upon alert. | Pass |
| Recommendation reduces uncertainty without adding noise | No, the problem lacks sufficient complexity, as clinicians already routinely identify malpositioned tubes within minutes. There does not exist true equipoise with this scenario. | Fail |
| **Checklist Item - Actionability** | **Assessment** | **Pass/Fail** |
| Action with an appropriate time frame? | Yes, tube repositioning can occur promptly, though this is already standard practice. | Pass |
| Alerts prompt action that otherwise would not occur | No. Clinicians already identify and act on malpositioned tubes without CDS assistance. | Fail |
| Non-actional alert rate is below the care team’s tolerance | Likely high. If the CDS generates false positives, such as flagging a tube that is 15 mm from the carina, it will breed clinician mistrust of the system and will lessen tolerance | Fail |
| **OVERALL** | **Fail - Problem is not complex enough. Clinicians already perform this task well without assistance, making CDS non-additive to clinical decision making.** | |

| **Supplemental Table 3: Example of a Problem Complex Enough but Not Actionable, Using the CAPE checklist** | | |
| --- | --- | --- |
| **Clinical Problem: Using recurrent neural network on time series data to predict the need for intubation in the next 48 hours in a recovering septic shock patient** | | |
| **Checklist Item - Complexity** | **Assessment** | **Pass/Fail** |
| Clinical decision and success metric ≤1 sentence | Predict the need for intubation in 48 hours in patients 1 week following the diagnosis of septic shock to enable early respiratory support escalation and avoid intubation | Pass |
| Success metric measurable within meaningful horizon | Intubation is a binary outcome within a defined and observable horizon of 48 hours | Pass |
| Recommendation reduces uncertainty without adding noise | There is significant variation in practice patterns for determination of emergent/unplanned intubations, indicating genuine uncertainty. | Pass |
| **Checklist Item - Actionability** | **Assessment** | **Pass/Fail** |
| Action with an appropriate time frame? | This is unclear. What is the appropriate action to take? Should we increase monitoring? Should we preemptively change respiratory support? | Fail |
| Alerts prompt action that otherwise would not occur | No, the patient would already be in the ICU with continuous monitoring. Most predictive factors are available to the ICU team. | Fail |
| Non-actional alert rate is below the care team’s tolerance | Unclear, but unlikely given the concerns brought about in items 1 and 2. | Fail |
| **OVERALL** | **Fail - While this problem has the appropriate complexity, it fails on actionability. There is no clear action pathway that differs from standard ICU care, making the CDS non-additive.** |  |

| **Supplemental Table 4: Example of a “Goldilocks” and Actionable Problem that Passes the CAPE checklist** | | |
| --- | --- | --- |
| **Clinical Problem: Using recurrent neural network on time series data to predict passing a spontaneous breathing trial (SBT)^1^ using pre-specified criteria** | | |
| **Checklist Item - Complexity** | **Assessment** | **Pass/Fail** |
| Clinical decision and success metric ≤1 sentence | Predicting the probability of passing the SBT will inform timing of SBT initiation. | Pass |
| Success metric measurable within meaningful horizon | Passing of failing the SBT can be observed shortly after model prediction. | Pass |
| Recommendation reduces uncertainty without adding noise | There is uncertainty regarding exactly when to initiate SBTs between providers. The CDS may reduce uncertainty by predicting SBT success. | Pass |
| **Checklist Item - Actionability** | **Assessment** | **Pass/Fail** |
| Action with an appropriate time frame? | The decision to initiate an SBT can occur almost immediately after model prediction. | Pass |
| Alerts prompt action that otherwise would not occur | The care team may not have initiated an SBT until later, routine clinical assessment. | Pass |
| Non-actional alert rate is below the care team’s tolerance | Likely low if appropriate thresholds are set for SBT passage. If calibrated correctly, the model would be unlikely to predict SBT passage when clinically unlikely. | Pass |
| **OVERALL** | **Pass – Problem selection has optimal complexity and leads to actionable output** |  |
| **^1^**Spontaneous breathing trials are often performed during as patients are weaning from mechanical ventilation to test the patient’s readiness for extubation. The test is typically performed by setting the ventilator to low ventilator support settings for a time-limited period and monitoring the patient’s physiologic response. The clinical problem this model attempts to address is *when* to initiate a spontaneous breathing trial. | | |
